# Fermented Sprouts of *Codonopsis lanceolata* Suppress LPS-Induced Inflammatory Responses by Inhibiting NF-κB Signaling Pathway in RAW 264.7 Macrophages and CD1 Mice

**DOI:** 10.3390/pharmaceutics15071793

**Published:** 2023-06-22

**Authors:** Seung-Hyuk Choi, So-Yeon Kim, Kyeong-Min Kim, Tamanna Jahan Mony, Ho Jung Bae, Min Seok Kim, Chan Ho Lee, Sun-Eun Choi, Sang Ho Lee, Se Jin Park

**Affiliations:** 1 Department of Food Biotechnology and Environmental Science, Kangwon National University, Chuncheon 24341, Republic of Korea; chltmdgur96@kangwon.ac.kr (S.-H.C.); ykims95@kangwon.ac.kr (S.-Y.K.); kasbai@kangwon.ac.kr (K.-M.K.); 2 Agriculture and Life Science Research Institute, Kangwon National University, Chuncheon 24341, Republic of Korea; tjmonycvasu@gmail.com (T.J.M.); baehj321@kangwon.ac.kr (H.J.B.); 3 Department of Forest Biomaterials Engineering, College of Forest and Environmental Sciences, Kangwon National University, Chuncheon 24341, Republic of Korea; ms23217@naver.com (M.S.K.); lgh4107@naver.com (C.H.L.); oregonin@kangwon.ac.kr (S.-E.C.); 4 College of Pharmacy, Jeju National University, Jeju 63243, Republic of Korea; 5 School of Natural Resources and Environmental Sciences, Kangwon National University, Chuncheon 24341, Republic of Korea

**Keywords:** *Codonopsis lanceolata*, lipopolysaccharide, sepsis, inflammation, fermentation

## Abstract

The interest in bioconversion through fermentation of sprouts produced in smart farms is increasing due to their potential health benefits. *Codonopsis lanceolata* (CL) is reported to alleviate inflammatory conditions, but much research is still needed to determine which types and parts of CL are most effective. This study investigated the anti-inflammatory effects of a fermented extract of CL sprouts’ aerial part (F-CSA) against LPS-stimulated RAW 264.7 macrophages and mice. In the screening test, F-CSA showed the most substantial anti-inflammatory effect among several samples, containing the highest total flavonoids, tannins, and polyphenols. UPLC-ESI-Q/TOF-MS and HPLC analysis revealed that F-CSA had the highest amount of luteolin among all the CL samples analyzed. F-CSA reduced the release of inflammatory cytokines and mediators such as NO and PGE_2_ by inhibiting the expression levels of iNOS and COX-2 in LPS-stimulated macrophages. Further, we found that the anti-inflammatory effects of F-CSA were mediated by inhibiting the JNK/NF-κB signaling pathway. Moreover, F-CSA improved survival rates and reduced plasma levels of NO and IL-6 in CD1 mice stimulated with LPS. These findings suggest that F-CSA, which contains luteolin, can alleviate inflammation in LPS-induced RAW 264.7 cells and a CD1 mouse model by inhibiting the JNK/NF-κB signaling pathways.

## 1. Introduction

Inflammation is a vital mechanism for protecting the body against harmful stimuli, but it can also lead to life-threatening organ dysfunction and critical illness [1]. Systemic inflammatory response syndrome can lead to a dysregulated cytokine storm, which can cause a massive inflammatory cascade, resulting in even death. Sepsis, a systemic inflammatory response syndrome caused by infection, is a physiological condition that involves a progressive imbalance between pro-inflammatory and anti-inflammatory responses in the body. Sepsis is characterized by accompanying end-organ failures and hemodynamic instability. Although there have been significant improvements in understanding the pathophysiology of acute systemic inflammatory responses, there is still no definitive therapeutic agent for treatment. A wide range of anti-inflammatory drugs are used to manage inflammatory responses [2]. Nonsteroidal anti-inflammatory drugs (NSAIDs) can alleviate symptoms associated with various medical conditions, such as tendinitis, arthritis, and other inflammatory responses [3]. The potential adverse effects of frequent use and high doses of NSAIDs, such as bleeding risk, kidney problems, and heart disease, are a cause for concern [4]. Moreover, the COVID-19 pandemic has increased the demand for functional foods and medications to enhance the body’s defense system [5]. Health functional foods, such as ginseng, known to be effective in boosting immunity, are attracting attention [6]. Furthermore, natural products offer advantages such as anti-oxidative and anti-inflammatory properties, rendering them a valuable resource for diverse therapeutic fields such as cancer, diabetes, and infectious diseases [7,8,9]. Although there have been reports of some side effects resulting from overuse and misuse, natural products can replace conventional anti-inflammatory drugs by providing a greater spectrum of pharmacological activities and lower toxicity [10].

*Codonopsis lanceolata* (CL) is traditionally utilized as a medicinal plant in East Asia, primarily in Korea, Japan, and China [11]. Especially, the root of CL has a popular and high value for the therapeutic purposes of asthma, cough, and psychoneurosis [12,13]. Furthermore, numerous studies support that CL positively affects the body, including antioxidant, hepatoprotective, anti-inflammatory, and anticancer effects [14,15,16,17]. CL comprises various bioactive components, such as flavonoids, polyphenols, tannins, and saponins [18]. These components can be enhanced through factors such as the collection part (leaf or root) and manufacturing processes such as fermentation. Furthermore, a study has indicated that sprout plants offer potential benefits in reducing risks associated with inflammation, cytotoxicity, and oxidative stress [19]. Additionally, active substances derived from fermented natural products have been shown to have significant anti-inflammatory effects and immune-regulating properties [20]. Previous studies have also reported that fermented basil and mint demonstrate improved bioavailability compared to their fresh counterparts [21]. In recent decades, numerous research and review papers have been released discussing the anti-inflammatory properties of various plants, such as CL [22,23]. However, research on the fermented or sprouted forms of CL is limited. Additionally, further investigations are required to determine which parts of the plant exhibit the most potent anti-inflammatory effects.

Fermented foods are an important type of cuisine in almost every culture worldwide, mainly due to the surging interest in gastrointestinal health and the health benefits of fermented foods [24]. The food fermentation process improves certain foods’ flavor, texture, aroma, and nutrition and the digestibility and availability of nutrients. *Aspergillus oryzae* is one of the most important fungi used in food fermentation, including the production of soybean paste and soy sauce [25]. Furthermore, *A. oryzae* has been used for a long time due to its involvement in the production of kojic acid, and it obtained safety approvals from the FDA and WHO. This fungus helps produce enzymes such as proteases and amylases, which break down proteins and starches into amino acids and sugars [26]. Therefore, fermentation by *A. oryzae* contributes to a smooth digestive process. Furthermore, it has been reported that the fermentation of *A. oryzae* can increase metabolites such as flavonoids and polyphenols [27]. 

Therefore, we compared the anti-inflammatory efficacy of part-specific and fermented extracts to explore the properties of CL enhanced by the fermentation process. In particular, we analyzed the regulatory signaling pathways and potential mechanisms underlying the effects of fermented CL sprout aerial part (F-CSA) on LPS-induced inflammatory responses in RAW 264.7 macrophages and a CD1 mouse model.

## 2. Materials and Methods

### 2.1. Animal

CD1 male mice (ICR mice, five weeks old) were supplied by Orient Bio (Seongnam, Republic of Korea). In each group, six mice were housed per cage. Animals were housed in laboratory animal solid feed (2018S; Envigo, Madison, WI, USA) and a water-supply controlled environment (rearing room temperature is 23 ± 2 °C, humidity is 60 ± 10%, and lighting is left at 12 h). The animal experiment was approved by the Kangwon National University Animal Experiment Ethics Committee (KW-200904-1).

### 2.2. Materials

The Folin–Ciocalteu phenol reagent, gallic acid, sodium hydroxide, catechin, sodium carbonate, sodium nitrite, aluminum chloride, polyvinyl polypyrrolidone (PVPP), potassium persulfate, lipopolysaccharide from *Escherichia coli* O127:B8 (LPS), 3-(4,5-dimethylthiazol-2-yl)-2,5-diphenyltetrazolium bromide (MTT), and Griess reagent were obtained from Sigma–Aldrich Co. (St. Louis, MO, USA). Dulbecco’s modified Eagle’s medium (DMEM), penicillin–streptomycin (P/S), and DEPC water were purchased from Welgene (Gyeongsan, Republic of Korea). In contrast, the fetal bovine serum (FBS) was purchased from Atlas Biologicals (Fort Collins, CO, USA). RNAiso Plus was obtained from Takara Bio Inc. (Kusatsu, Japan), and the primers for TNF-α, IL-6, IL-1β, iNOS, COX-2, and β-actin were obtained from Integrated DNA Technologies (Coralville, IA, USA). TransScript All-in-One First-Strand cDNA synthesis SuperMix for qPCR (one-step gDNA removal) was purchased from TransGen Biotech Co. (Beijing, China). At the same time, the PowerSYBR^®^ Green PCR Master Mix was obtained from Thermo Fisher Scientific (Rockford, IL, USA). The ELISA kit for prostaglandin E_2_ (PGE_2_) was obtained from R&D Systems (Minneapolis, MN, USA), and the interleukin-6 (IL-6) ELISA kit was purchased from Abcam (Cambridge, UK). Antibodies against p38, JNK, ERK, p65, phosphorylated p38 (p-p38), phosphorylated JNK (p-JNK), phosphorylated ERK (p-ERK), phosphorylated p65 (p-p65), iNOS, COX-2, and β-actin were purchased from Cell Signaling Technology (Danvers, MA, USA). All other materials and reagents used were of the highest available quality.

### 2.3. Preparation of Extracts and Samples

Each CL sample was donated by Danong Co., Ltd. (Wonju, Republic of Korea). The plant specimen was authenticated, and a plant identification certificate was provided by Dr. Jang (Ph.D. of Botany in Kangwon National University). The LED-based smart farm facility cultivated the CL sprout for 20 days. The temperature of the smart farm was maintained at 19 °C (night/8 h)~24 °C (day/16 h). The light sources were blue and red LEDs (24 V): the wavelength of blue light was set to 430 to 500 nm; the wavelength of red light was set to 600 to 660 nm (peak 631.0 nm). Water was sprayed on the roots for 30 s every 90 min to grow the CL sprout. After obtaining the CL sprout, it was divided into aerial parts and roots for further experiments.

The fermentation of CL was carried out by Baekcheon Biotech (Yanggu, Republic of Korea). For fermentation, *Aspergillus oryzae* was used as the strain. The culture temperature was 36 °C, the culture humidity was 50~80%, and the culture time was 72 h. The cultured semifinished products were selected by a sieve, placed in a drying tray, and dried at a temperature of 40~50 °C for 4 h to maintain a water content below 8%.

All samples were extracted with 70% ethanol twice for 2 h in an ultrasonic bath to obtain the ethanolic extract. The solution was filtered and concentrated using a rotary vacuum evaporator. After that, lyophilization was performed using a FD-5 N machine (Eyela, Tokyo, Japan). The extract was stored at −20 °C, and the yield of each six CL sample is represented in Table 1.

### 2.4. UPLC-ESI-QTOF-MS Analysis

Ultra-performance liquid chromatography (UPLC)–electrospray ionization (ESI)–quadrupole time-of-flight (QTOF) mass spectrometry (MS) analysis was carried out on an Acquity UPLC system (Waters Corp., Milford, MA, USA) coupled with a Waters Xevo G2 QTOF MS system (Waters Corp., Manchester, UK) at the Chuncheon Center of Korea Basic Science Institute (KBSI). The Waters Acquity UPLC BEH C18 column (2.1 mm × 50 mm, 1.7 μm) was used with the mobile phase consisting of water with 0.1% formic acid and acetonitrile with 0.1% formic acid. The gradient was 10–90% B (0–14 min). The injection volume was 2 µL, and the flow rate was 0.4 mL/min. The TOF MS detection was performed using modern MSE data acquisition in negative ESI ionization modes. To conduct the tandem mass experiment, the negative electrospray ionization (ESI) method was employed, and data were collected across a range of *m*/*z* values from 100 to 1600. The source temperature was adjusted to 120 °C, while the desolvation temperature was maintained at 350 °C with a desolvation gas flow rate of 800 L/h. To ensure accuracy, leucine enkephalin at a concentration of 200 pg/μL was used as the lock mass. The capillary and cone voltage were set to 2.5 kV and 45 V, respectively. Finally, collision energy was ramped up from 15 to 40 eV during the experiment. The compounds were identified using the in-house library (UNIFI 1.8, Waters Corp., Milford, MA, USA), and the instrument was managed by Waters Masslynx software (v 4.1).

### 2.5. Quantitative Analysis of Luteolin and Kaempferol Using HPLC

Quantitative analysis of the luteolin and kaempferol of six CL samples was performed using a reverse HPLC system. HPLC analysis was performed with an HPLC instrument (Waters Alliance e2695 Separations Module, Milford, MA, USA) equipped with an ultraviolet–visible spectrophotometric detector (Waters 2489 UV/Vis Detector, USA), a pump, and an auto-sampler with a YMC Pack Pro C18 column (4.6 × 250 mm, 5 μm). The injection volume was 10 μL, and the flow rate was set to 1.0 mL/min. The wavelength of the UV/Vis detector was set to 270 nm. The analysis was conducted via the gradient method with 0.1% trifluoroacetic acid in water (A) and acetonitrile (B). The elution system was as follows: 0–10 min 83% (A); 40 min 30% (A); 41–45 min 0% (A); 50 min 83% (A); 60 min 83% (A). The temperature of the column was maintained at 30 °C. The reference standards for luteolin and kaempferol were obtained from the Natural Product Institute of Science and Technology (Anseong, Republic of Korea). The standard solutions were prepared by dissolving two compounds in MeOH at a concentration of 1 mg/mL. The working solution used to construct the calibration curve was prepared by continuous dilution to the desired concentration. The six CL samples, weighing 10 mg, were dissolved in 1 mL of methanol. Both the standard and six CL sample solutions were passed through a 0.45 μm PVDF membrane filter before injection. Subsequently, the luteolin and kaempferol concentrations were determined from the calibration curve using peak area (Y) and concentration (X, μg/10 μL) as well as calculating the average mean (*n* = 5) ± standard deviation (SD) (Table 2).

### 2.6. Determination of Total Polyphenols

To determine the total phenolic compounds in the CL extracts, we used the Folin–Ciocalteu colorimetric method described by Singleton and Rossi [28]. For this, 0.50 mL of the CL extract in methanol (1 mg/mL) was mixed with 0.75 mL of the Folin–Ciocalteu reagent, and then 0.4 mL of saturated sodium carbonate solution was added. The resulting mixture was kept in the dark for 2 h at room temperature, and then the absorbance was measured at 760 nm using a microplate reader (SpectraMax 190, Molecular Devices, San Jose, CA, USA). This process was repeated three times. The results were expressed as milligram gallic acid equivalents per gram of dry weight of the respective formulation extract (mg GAE/g extract of the formulation), with SD for the three replicate analyses.

### 2.7. Determination of Total Tannin

Total phenolics include tannin and non-tannin phenolics. To determine the non-tannin phenolics, a process was followed. First, 500 µL of the extract and 500 µL of distilled water were added to 2 mL Eppendorf tubes that contained 100 mg of polyvinylpolypyrrolidone (PVPP). These tubes were then incubated at 4 °C for 4 h and centrifuged at 3000 rpm for 10 min at 4 °C. After centrifugation, the supernatant was collected, and the non-tannin phenolics were measured using the same method to determine the total phenolics. The tannin content was determined by subtracting the non-tannin phenolics from the total phenolics [29]. The results were reported in milligrams of gallic acid equivalents per gram of dry weight of the extract, along with the SD for three replicate analyses.

### 2.8. Determination of Total Flavonoid and Saponin

A colorimetric method was used to determine the total flavonoid compounds in the CL extracts [30]. Firstly, 400 μL of the formulation extracts in methanol (1 mg/mL) were mixed with 30 μL of 5% sodium nitrite and left for 5 min at room temperature. Subsequently, 30 μL of 10% AlCl_3_·6H_2_O solution were added, and the mixture was left for another 5 min. Then, 200 μL of 1 M NaOH were added, and the final reaction mixture was diluted to 1 mL with distilled water and left in the dark for 15 min at room temperature. The absorbance was measured at 510 nm using a microplate reader. A calibration curve was prepared using catechin. The total flavonoid content was expressed as milligrams of catechin equivalent per gram of dry weight of the formulation extract (mg CE/g extract of the formulation), with the standard deviation reported for three replicates.

To determine the total saponin compounds in the CL extracts, a colorimetric method [31] was used. First, 250 µL of 1000 µg/mL extracts, 250 µL of 8% vanillin, and 2.5 mL of 72% sulfuric acid were mixed in a glass. The solution was then sonicated for 10 min at 60 °C, and 200 µL of the mixture was placed in each well. The solution was then placed in ice water. The standard calibration curve absorbance was measured at 510 nm using a microplate reader (SpectraMax 190, Molecular Devices, San Jose, CA, USA) by reacting the same way as the diosgenin sample. The total saponin content was expressed as milligrams of diosgenin equivalent per gram dry weight of the formulation extract (mg DE/g extract) with ± SD for three replicates.

### 2.9. Cell Culture

RAW 264.7 cells were obtained from the Korean Cell Line Bank (KCLB) and were mouse-derived macrophages. The cells were grown in Dulbecco’s modified Eagle’s medium (DMEM) supplemented with 100 units/mL penicillin–streptomycin (P/S) and 10% fetal bovine serum (FBS). The cells were incubated at 37 °C in a 5% CO_2_ incubator and were subcultured every two days.

### 2.10. Cell Viability Analysis

RAW 264.7 cells were seeded at 2 × 10^5^ cells/well in a 96-well plate and incubated for 24 h in a 5% CO_2_ incubator at 37 °C. Cell viability was assessed using the MTT assay. After the incubation, MTT solution was added and incubated for 4 h at 37 °C. The formazan crystals formed were dissolved in ethanol, and the optical density was measured at 540 nm using a microplate reader (SpectraMax 190, Molecular Devices, San Jose, CA, USA).

### 2.11. Measurement of Nitric Oxide

RAW 264.7 cells (2 × 10^6^ cells/well) were pretreated with each sample for the screening test (500 and 1000 μg/mL) or F-CSA (125–1000 μg/mL) for 1 h, after which they were stimulated with LPS (1 μg/mL) for 24 h. Nitrite accumulation in the culture medium as an indicator of nitric oxide (NO) production was measured using the Griess reagent [32]. The cell supernatant was mixed 1:1 with 100 μL of Griess reagent for 10 min. The optical density was measured using a microplate reader (SpectraMax, Molecular Devices, San Jose, CA, USA) at 540 nm. The sodium nitrite (NaNO_2_) standard graph was used to calculate the NO concentration.

### 2.12. mRNA Expression Analysis Using RT-qPCR

The mRNA expression levels of iNOS, COX-2, IL-6, IL-1β, and TNF-α were detected using real-time quantitative polymerase chain reaction (RT-qPCR) [33]. F-CSA pretreatment and LPS-stimulated RAW 264.7 cells were dissolved using RNAiso plus to extract total RNA. An amount of 1 μg total RNA was reverse transcripted to cDNA using All-in-One First-Strand cDNA Synthesis SuperMix. For the RT-qPCR, gene-specific primers (Table 3) were used with POWER SYBR Green PCR master mix, and it conducted on a QuantStudio 3 (Applied Biosystems, Foster City, CA, USA) system. The conditions of RT-qPCR consisted of 40 cycles: 15 s at 95 °C, 20 s at 57 °C, and 40 s at 72 °C, followed by 15 s at 95 °C, 1 min at 60 °C, and 15 s at 95 °C.

### 2.13. Western Blot Analysis

The F-CSA pretreated and LPS (1 μg/mL)-stimulated RAW 264.7 cells were collected using a scraper and lysed using a lysis buffer (Jubiotech, Daejeon, Republic of Korea). The total protein was measured using the Bradford assay. A total of 20 μg protein was loaded onto 10% SDS-PAGE gels and then transferred to PVDF membranes. The membrane was then blocked with a blocking buffer (5% skimmed milk powder in 1X TBS containing 0.1% Tween-20) for 2 h. The membrane was incubated overnight at 4 °C with primary antibodies against various proteins, including p-ERK, p-p38, p-JNK, p-p65, ERK, p38, JNK, p65, iNOS, COX-2, and β-actin (all from Cell Signaling Technology, 1:1000). After washing the membrane, it was left to incubate for 2 h at room temperature (20 ± 5 °C) with a secondary antibody (Cell Signaling Technology, 1:1000). The membrane was developed using enhanced chemiluminescence, and the immunoblots were captured using a LAS-500 mini-imager (General Electric, Boston, MA, USA). The captured images were analyzed using ImageJ software, version 1.51j8 (LOCI, University of Wisconsin, Madison, WI, USA). The degree of phosphorylation was determined by calculating the ratio of the amount of phosphorylated protein to the total protein on the same membrane.

### 2.14. ELISA Analysis

The levels of PGE_2_ (R&D systems, Minneapolis, MN, USA) in culture medium or IL-6 (Abcam, Cambridge, UK) in plasma were quantified using ELISA kits according to the manufacturer’s protocols.

### 2.15. LPS-Induced Septic Shock Model

To investigate various physiological effects of F-CSA in LPS-induced septic shock mice (12 mice/group) and normal mice (6 mice), the mice were one-time orally administered 50, 100, or 200 mg/kg F-CSA or a vehicle (0.9% saline) as a control. To cause septic shock in the mice, LPS 25 mg/kg was intraperitoneally injected. The survival rate was observed at 12 h intervals for 72 h. For biochemical analysis (4 mice/group), mice were sacrificed 12 h after LPS injection, and blood samples were collected to measure the levels of NO and IL-6 in the plasma.

### 2.16. Statistical Analyses

The data was analyzed using GraphPad Prism Version 8 software (GraphPad, La Jolla, CA, USA). The mean value and standard error of the mean (S.E.M) were used to express the data. One-way analysis of variance (ANOVA) was performed to analyze the data, followed by the Student–Newman–Keuls test for multiple comparisons. A statistical value of *p* < 0.05 was considered to be significant.

## 3. Results

### 3.1. Effects of Six CL Samples on Cell Viability and LPS-Induced NO Production in RAW 264.7 Cells

**Figure 1:**
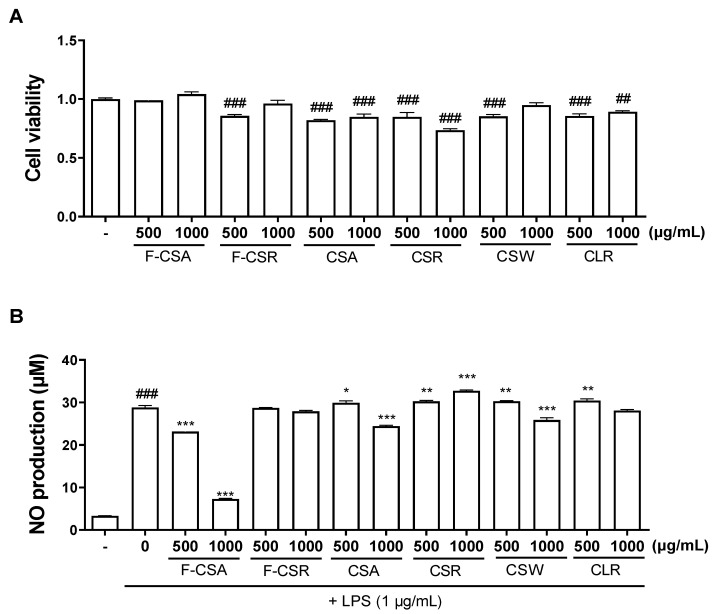
Effects of six CL samples on cell viability and LPS-induced NO production in RAW 264.7 cells. (**A**) Six CL samples were treated for 24 h, and cytotoxicity was measured using MTT assay. (**B**) Six CL samples were treated 1 h before LPS stimulation, and NO was quantified 24 h later with Griess reagent. F-CSA: Fermented *C. lanceolata* sprout aerial part; F-CSR: Fermented *C. lanceolata* sprout root zone; CSA: *C. lanceolata* sprout aerial part; CSR: *C. lanceolata* sprout root zone; CSW: *C. lanceolata* sprout whole plant; CLR: *C. lanceolata* root zone. *^##^ p* < 0.01, *^###^ p* < 0.001 compared to vehicle alone; * p < 0.05, ** p < 0.01, *** p < 0.001 compared to LPS alone.

First, we investigated the effects of each sample (F-CSA, F-CSR, CSA, CSR, CSW, CLR) on cell viability and LPS-induced NO production in RAW 264.7 cells. We found that all six samples did not show cytotoxicity up to 1000 µg/mL (Figure 1A). Moreover, we observed that only F-CSA exhibited significant inhibition of LPS-induced NO (Figure 1B). These results indicate that F-CSA has potential anti-inflammatory efficacy among the six samples.

### 3.2. Contents Analysis of Polyphenols, Tannins, Flavonoids, and Saponins in Six CL Samples

The contents of total polyphenols, tannins, flavonoids, and saponins were measured (Table 4). The total polyphenol and tannin content of each of the six CL samples were determined as gallic acid equivalents, with F-CSA exhibiting the highest total polyphenol (57.00 mg GAE/g) and total tannin (36.27 mg GAE/g) among the six CL samples. The total flavonoids were measured only in aerial part samples (F-CSA and CSA), which were determined as catechin acid equivalents. The total flavonoid content of F-CSA (26.17 mg CE/g) is higher than the non-fermented sample (CSA). However, the amount of total saponin was detected in all samples, and the roots zone samples (CSR and CLR) were measured to contain particularly large amounts. The content of total saponins was found to be higher in the underground portion of the plant, which may be due to differences in the extraction method used for plant materials. These results indicate that F-CSA shows an increased content of polyphenols, tannins, and flavonoids, suggesting that CSA may enhance the amounts of bioactive compounds during fermentation.

### 3.3. Qualitative Analysis of F-CSA Using UPLC-ESI-Q/TOF-MS Analysis

**Figure 2:**
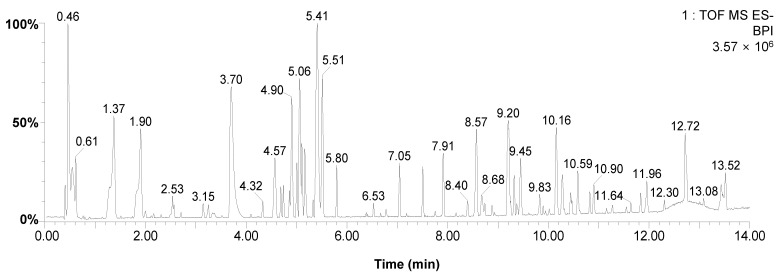
Base peak chromatograms of the F-CSA by UPLC-ESI-Q/TOF-MS in negative ion mode.

Based on a comparative study of six CL samples, we found that F-CSA has the potential for anti-inflammatory effects. In addition, we found that F-CSA has increased the contents of bioactive substances including total phenolic, tannin, and flavonoid. Therefore, we next conducted UPLC-ESI-Q/TOF-MS to identify F-CSA’s major components qualitatively. Optimized chromatographic conditions were achieved by testing the mobile phase, elution program, and mass spectrometry needs. UPLC-ESI-Q/TOF-MS identified the target compositions in negative ionization mode (Figure 2). All target analytes in the negative ESI experiments had the deprotonated molecule [M − H]^−^ in their MS/MS spectra, with an accuracy of 4.0 ppm. The analysis revealed the presence of 64 compounds in F-CSA, belonging to various subclasses such as phenolic compounds, flavonoids, terpenes, and fatty acids. Among these, 17 compounds were tentatively identified based on their correspondence with previously reported literature, while the remaining compounds were classified as unknown (Table 5). The specific details of the identified compounds can be found in Table 5. Flavonoid components such as luteolin (C_15_H_10_O_6_) and kaempferol (C_15_H_10_O_6_) detected in F-CSA have been reported to have anti-inflammatory effects [34,35]. Previous studies reported that both luteolin and kaempferol downregulate MAPK/NF-κB-mediated inflammation [36,37]. Luteolin has been reported to attenuate LPS-induced organ damage and myocardial injury [38,39]. In addition, kaempferol has been reported to potentially inhibit oxidative tissue injury and pulmonary inflammatory response [40]. Therefore, we speculated that luteolin and kaempferol are the main components of F-CSA that show the anti-inflammatory effect.

### 3.4. Quantitative Analysis of Luteolin and Kaempferol in Six CL Samples

**Figure 3:**
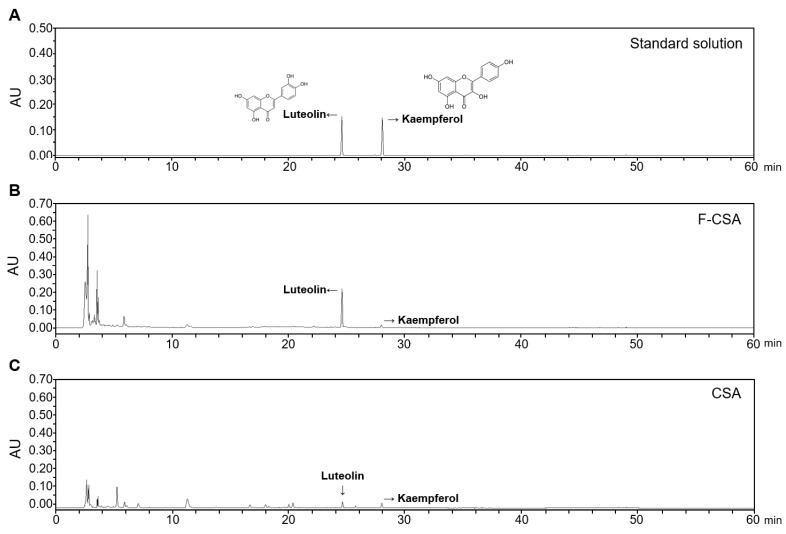
Representative HPLC chromatographs of (**A**) standard solution, (**B**) F-CSA, and (**C**) CSA.

In the quantitative analysis of six CL samples using HPLC, two compounds, luteolin and kaempferol, were quantified (Table 6). Notably, we observed the presence of luteolin and kaempferol exclusively in the aerial parts, F-CSA and CSA (Figure 3). The F-CSA contained a high content of luteolin (7.83 ± 0.16 mg/g) and also showed the presence of kaempferol (1.09 ± 0.07 mg/g). Both luteolin (1.71 ± 0.36 mg/g) and kaempferol (1.65 ± 0.27 mg/g) were also detected in CSA. In CSW, both compounds were detected in trace amounts, making quantitative analysis difficult. Neither luteolin nor kaempferol were detected in CLR, CSR, and F-CSR, the root parts. These results are consistent with the flavonoid content analysis in six CL samples (Table 4). These findings suggest that fermentation of CSA enhances the contents of flavonoids, particularly luteolin.

### 3.5. Effects of F-CSA on Cell Viability and LPS-Induced Inflammatory Reactions in RAW 264.7 Macrophages

**Figure 4:**
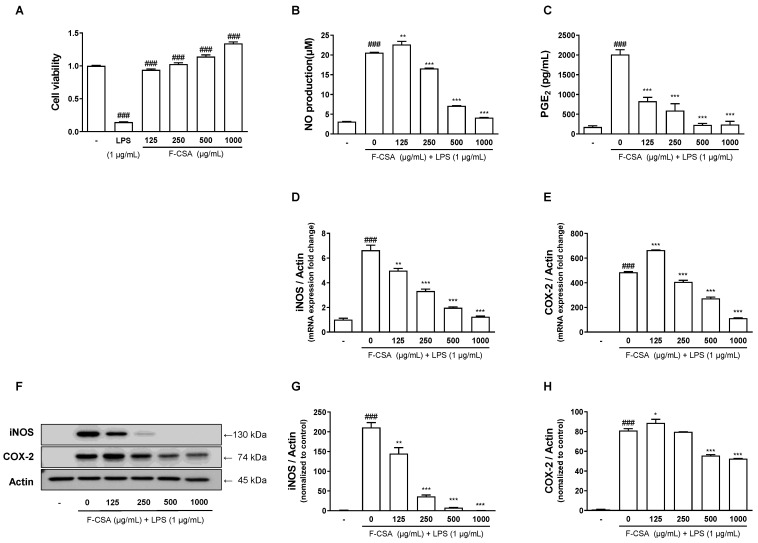
Anti-inflammatory effect of F-CSA in LPS-induced RAW 264.7 macrophages. (**A**) Cytotoxicity of F-CSA is measured by cell viability using the MTT assay. The production of (**B**) NO and (**C**) PGE_2_ was determined 24 h after F-CSA pretreatment and LPS stimulation. The expression of (**D**) iNOS and (**E**) COX-2 was determined using RT-qPCR. (**F**–**H**) The relative protein levels are represented normalized to control. *^###^ p* < 0.001 compared to vehicle; * p < 0.05, ** p < 0.01, *** p < 0.001 compared to LPS alone.

Luteolin has been reported to reduce LPS-induced inflammation by inhibiting the MAPK and NF-κB signaling pathways [41]. Therefore, we investigate whether F-CSA had anti-inflammatory effects in LPS-induced macrophages. To determine its cytotoxicity, an MTT assay was performed in RAW 264.7 macrophages. The cells were treated with various concentrations of F-CSA (125–1000 μg/mL) for 24 h, and cell viability was analyzed. F-CSA was found to be non-toxic to cells at concentrations below 1000 μg/mL, exhibiting a significant increase compared to the vehicle-treated group (Figure 4A).

Next, we evaluated the anti-inflammatory effects of F-CSA under 1000 μg/mL in LPS-induced RAW 264.7 cells. The cells were pretreated with F-CSA for 1 h before being treated with LPS (1 μg/mL) for 24 h. We observed that F-CSA dose-dependently inhibited LPS-induced NO production (Figure 4B). The mRNA and protein expression levels of iNOS, an enzyme involved in NO production, were measured using RT-qPCR and Western blotting, respectively. F-CSA significantly decreased NO production by inhibiting the expression of iNOS at both mRNA and protein levels, suggesting that it reduced LPS-stimulated NO production in RAW 264.7 macrophages (Figure 4D,G). Furthermore, we found that F-CSA decreased the generation of PGE_2_ dose-dependently (Figure 4C). In addition, the mRNA expression of COX-2, an enzyme responsible for synthesizing PGE_2_, was also significantly reduced at F-CSA concentrations of 250–1000 μg/mL (Figure 4E,H). These findings suggest that F-CSA can regulate the production of PGE_2_ by suppressing the expression of COX-2, which could have anti-inflammatory effects. Altogether, these findings indicate that F-CSA has the potential as an anti-inflammatory agent.

### 3.6. The Effect of F-CSA on Expression of Pro-Inflammatory Cytokines in LPS-Stimulated RAW 264.7 Macrophages

**Figure 5:**
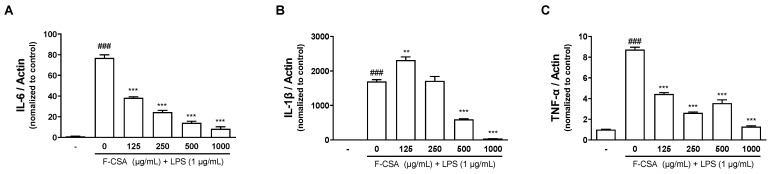
Inhibitory effects of F-CSA on LPS-induced inflammatory cytokines. The mRNA expression of (**A**) IL-6, (**B**) IL-1β, and (**C**) TNF-α was determined using RT-qPCR and normalized to control. *^###^ p* < 0.001 compared to vehicle; ** p < 0.01, *** p < 0.001 compared to LPS alone.

During excessive inflammatory responses, immune cells secrete inflammatory cytokines that induce various inflammatory reactions [42]. To address this issue, we investigated the inhibitory effects of F-CSA on the production and expression of inflammatory cytokines, including IL-6, IL-1β, and TNF-α in LPS-stimulated RAW 264.7 macrophages. F-CSA significantly dose-dependently reduced the mRNA expression of IL-6, IL-1β, and TNF-α. Moreover, F-CSA 1000 µg/mL significantly reduced the mRNA expression of inflammatory cytokines to a level similar to that in the vehicle treatment group (Figure 5).

### 3.7. The Effect of F-CSA on the JNK/NF-κB Signaling Pathway in LPS-Stimulated RAW 264.7 Macrophages

**Figure 6:**
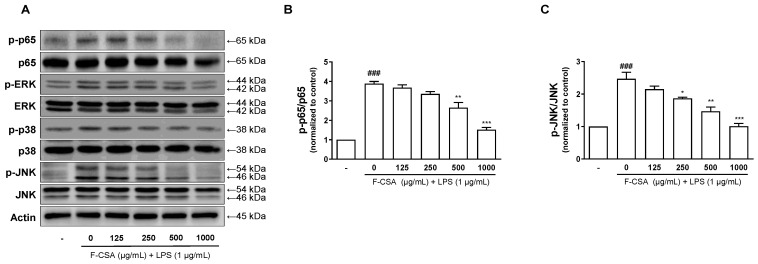
Attenuation of MAPK phosphorylation and NF-κB signaling by F-CSA. (**A**) The expression of phosphorylated p65 (p-p65), ERK (p-ERK), p38 (p-p38), JNK (p-JNK), p65, ERK, p38, JNK. The phosphorylation level of (**B**) p65 and (**C**) JNK was normalized to control. *^###^ p* < 0.001 compared to vehicle; * p < 0.05, ** p < 0.01, *** p < 0.001 compared to LPS alone.

When LPS binds to TLR4, it activates transcription inducers such as NF-κB by facilitating phosphorylation [43]. It is well known that the MAPK/NF-κB phosphorylation pathway is responsible for the expression of inflammatory cytokines in LPS-stimulated RAW 264.7 cells [44]. After pretreatment with F-CSA for 1 h, the cells were stimulated with 1 μg/mL of LPS for 1 h. The cells were then collected to demonstrate that F-CSA has anti-inflammatory properties by regulating the MAPK/NF-κB pathway. In Figure 6A, we observed that LPS increased phosphorylation of p65, ERK, p38, and JNK by Western blot analysis. Furthermore, our quantitative results revealed that LPS-induced p65 and JNK phosphorylation was suppressed depending on the concentration of F-CSA (Figure 6B,C). Notably, F-CSA had no effect on the LPS-induced increase in ERK and p38 phosphorylation. Therefore, these results suggest that F-CSA might exhibit anti-inflammatory effects via inhibition of the JNK/NF-κB pathway.

### 3.8. Effects of F-CSA on Survival Rate and the Levels of NO and IL-6 in LPS-Stimulated Septic Shock

**Figure 7:**
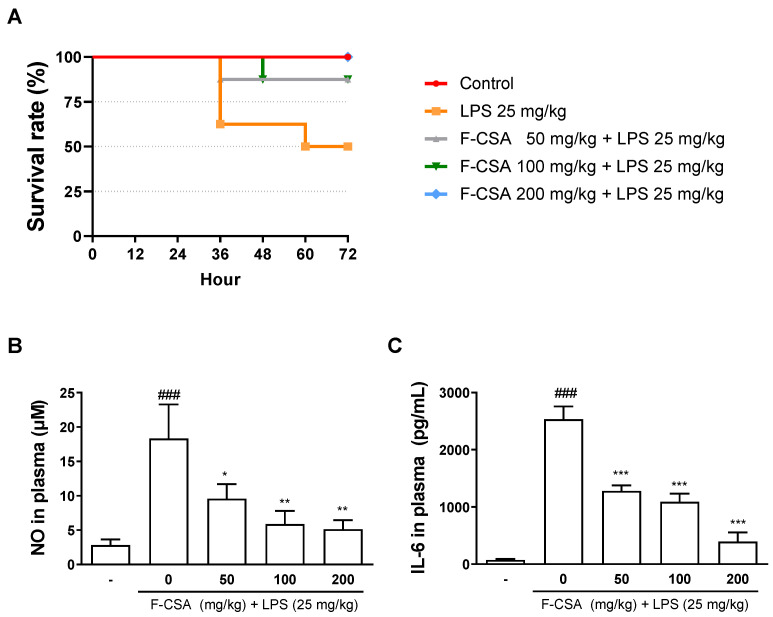
Antiseptic effect of F-CSA on LPS-induced septic shock mice. F-CSA (50, 100, and 200 mg/kg) was administered orally once 1 h before LPS injection (25 mg/kg, i.p.). (**A**) After injecting LPS, the survival rate was measured every 12 h for 3 days (*n* = 8). (**B**) The NO production and (**C**) level of IL-6 in plasma were determined 12 h after LPS injection (*n* = 4). *^###^ p* < 0.001 compared to control group; * p < 0.05, ** p < 0.01, *** p < 0.001 compared to LPS 25 mg/kg group.

We investigated the potential antiseptic effects of F-CSA, a substance with known anti-inflammatory properties, in a sepsis mouse model induced by intraperitoneal injection of LPS. Three doses of F-CSA (50, 100, and 200 mg/kg) were orally administered to the mice 1 h before LPS injection, and the survival rate was monitored at 12 h intervals. The LPS-treated group had the lowest survival rate (50%) after three days of LPS injection, while the F-CSA-treated group showed a dose-dependent increase in survival rate (from 87.5% to 100%) (Figure 7A). In addition, we investigated the effect of F-CSA on NO and IL-6, inflammatory mediator and cytokine, that can lead to death if produced excessively [45]. The plasma was collected 12 h after the LPS injection, and we observed a significant increase in the levels of NO and IL-6 in the plasma of the LPS-treated group. However, we observed that F-CSA dose-dependently reduced the levels of NO and IL-6 (Figure 7B,C). These findings suggest that F-CSA can attenuate LPS-induced septic shock in mice by inhibiting the production of NO and IL-6.

## 4. Discussion

In the present study, we examined the impact of F-CSA on inflammatory response in macrophages and septic shock mice. The study found that F-CSA had a positive effect on both. Specifically, when LPS-stimulated RAW 264.7 macrophages were treated with F-CSA, it was found to improve the inflammatory response. Additionally, our results showed that F-CSA is not cytotoxic using the MTT assay. It should be noted that the MTT assay is a colorimetric method that is sensitive to absorbance. Hence, this result was calculated after subtracting the color absorbance of F-CSA as a blank. However, it is essential to note that the color of F-CSA may have influenced this result and does not necessarily indicate cell proliferation. Moreover, administering F-CSA to mice with LPS-induced septic shock improved their survival rate and reduced their plasma levels of IL-6 and NO. These results suggest that F-CSA could be a promising dietary supplement for preventing septic shock.

*Codonopsis lanceolata* (CL) is a perennial herb that contains polyacetylenes, antioxidants, salicylate derivatives, and polysaccharides, which exert various biological effects in traditional medicine [11]. A smart farm, an indoor greenhouse, regulates environmental conditions such as temperature and humidity using information and communication technology (ICT). Smart farms offer the advantage of maximizing agricultural production per unit area and improving crop quality [46]. The growth of CL was carried out at Daily Farm in Hoengseong, which is fully equipped with LED smart farm facilities and is currently in production operation. Moreover, food fermentation is an effective way to increase digestibility and bioavailability. In particular, *A. oryzae*, which ferments soybeans, is known to increase metabolites such as flavonoids and polyphenols during the fermentation process [47]. In this study, we found that F-CSA improved the contents of active components, including flavonoids and polyphenols found in fruits, vegetables, and medicinal plants, compared to other CL samples. Notably, luteolin in F-CSA significantly increased by approximately 4.5-fold compared to its pre-fermentation, CSA, reaching approximately 7.83 ± 0.16 mg/g. Luteolin has various beneficial properties, including anti-inflammatory, antioxidant, anticancer, antidiabetic, and neuroprotective activities [38,48,49]. In addition, luteolin prevents liver injury caused by chemicals or drugs by inhibiting ROS, inflammation, and autophagy [50]. Moreover, luteolin reduced inflammatory cytokines such as IL-6 by regulating LPS-induced NF-κB [51]. Therefore, anti-inflammatory and antiseptic effects of F-CSA are exhibited by components such as luteolin, which are enhanced during fermentation [52].

LPS activates immune cells such as macrophages, monocytes, and neutrophils in the liver, spleen, and blood, which triggers the movement of mitogen-activated protein kinase (MAPK) [53]. MAPK is a group that includes extracellular signal-regulated kinase (ERK), p38, and c-JUN-terminal kinase (JNK). ERK is a crucial mediator during signal transduction in response to cellular stresses and various tissue injuries [54]. Previous studies also demonstrated that p38 was necessary to induce inflammatory gene expression [55]. Furthermore, JNK activated by extracellular stress is associated with inflammation and induces the production of inflammatory factors, including TNF-α and IL-6 [56,57]. Notably, phosphorylated by external stimuli such as LPS, these MAPKs cause phosphorylation of NF-κB, leading to inflammation. NF-κB is a transcription factor that regulates immunomodulation and plays a critical role in inflammatory reactions. The NF-κB signaling pathway comprises several members, including p65, which controls both physiological and pathological processes [58]. The transcription factor p65 relocates to the nucleus and promotes the release of different inflammatory factors, which are referred to as inflammatory cytokines, such as interleukin-6 (IL-6), tumor necrosis factor-α (TNF-α), and interleukin-1β (IL-1β) [59]. Pro-inflammatory cytokines, including IL-6, TNF-α, and IL-1β production, are vital for initiating and regulating immune responses to different inflammatory stimuli. In this study, we observed that F-CSA reduced the phosphorylation of JNK and the corresponding reduction in phosphorylation of NF-κB, inhibiting the expression of inflammatory factors that cause chronic inflammation. However, our results showed that F-CSA did not reduce the activation of ERK and p38 in LPS-induced RAW 264.7 macrophages. Therefore, it is considered that F-CSA reduces the expression of inflammatory cytokines by suppressing the activation of the JNK/NF-κB pathway in RAW 264.7 macrophages induced by LPS. Moreover, compared to the control group treated only with LPS, F-CSA showed a dose-dependent decrease in mRNA expression levels of TNF-α, IL-6, and IL-1β. Overall, these results suggested that F-CSA could potentially reduce the production of inflammatory cytokines induced by LPS.

Excessive activation of NF-κB increases the expression of inducible nitric oxide synthase (iNOS) and cyclooxygenase-2 (COX-2) enzymes that synthesize inflammatory agents [60]. iNOS synthesizes nitric oxide (NO), a type of reactive oxygen species (ROS). NO is critical as an inflammatory mediator and regulatory molecule in multiple physiological processes, including neurotransmission, and is indispensable for defending the host against pathogens [61]. However, unbalanced NO production, particularly in macrophages, can cause inflammation, cytotoxicity, and autoimmune disorders [62,63]. COX-2 is strongly associated with inflammation and is the primary inducible enzyme responsible for catalyzing the transformation of arachidonic acid into prostaglandins [64]. PGE_2_ is a widely recognized prostanoid generated in activated cells and tissues following COX-2 activation. Macrophages and keratinocytes are the primary sources of its release [65]. F-CSA demonstrated inhibitory effects on LPS-induced NO production. Therefore, it can be assumed that F-CSA reduces the mRNA and protein expression levels of iNOS and COX-2, inhibiting NO and PGE_2_.

Cytokines are released from the inflammatory site into the bloodstream, leading to a systemic inflammatory response syndrome. Excessive and persistent inflammation is worsened by impaired immune function and infection, which can cause sepsis or multiple organ dysfunction syndrome [66]. Especially, plasma levels of IL-6 are considered an important biomarker positively associated with septic shock in patients. Furthermore, patients who exhibit elevated levels of IL-6 have a lower survival rate than those with lower levels, prompting studies that aim to develop therapeutic agents such as anti-IL-6 antibodies [67,68,69]. The effectiveness of F-CSA in treating sepsis was evaluated using an LPS-induced mouse model in this study. F-CSA administered orally significantly reduced IL-6 and NO levels in the blood of septic mice after LPS treatment. Moreover, the survival rate of mice after LPS stimulation was significantly increased with oral F-CSA administration. These results suggest that elevated plasma IL-6 levels in LPS-induced septic shock were normalized with F-CSA, leading to an increase in the survival rate of mice. While F-CSA treatment reduced the expression of IL-1β and TNF-α in LPS-stimulated RAW 264.7 cells, these changes were not observed in mice with LPS-induced sepsis. Therefore, further studies are needed to confirm the effects of F-CSA treatment on various proinflammatory cytokines in mice with sepsis.

**Figure 8:**
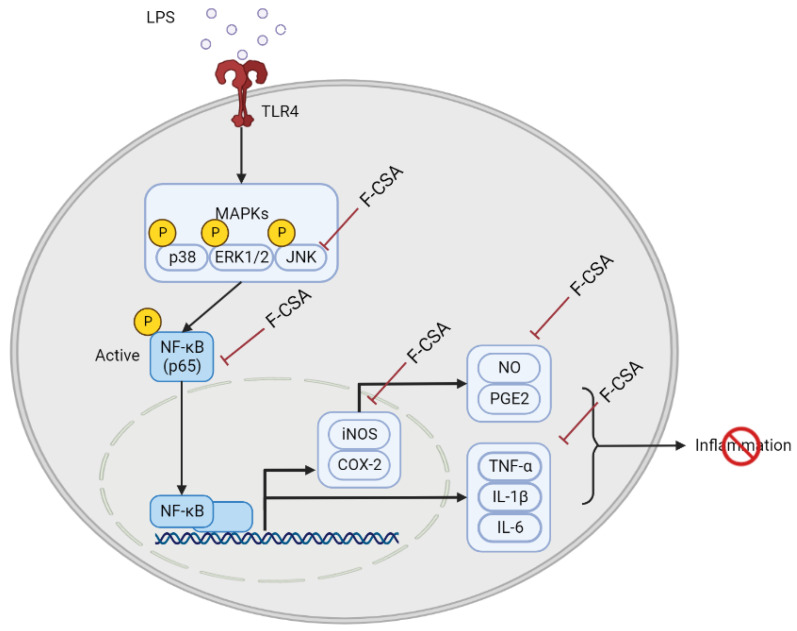
Proposed a potential mechanism of the anti-inflammatory effect of F-CSA. Pre-treatment with F-CSA inhibits the phosphorylation of JNK, which in turn blocks the translocation of NF-κB to the nucleus. This leads to a decrease in the production of various pro-inflammatory cytokines such as TNF-α, IL-1β, and IL-6, as well as chemokines and NO and PGE_2_, compared to LPS stimulation in the absence of F-CSA pre-treatment. This figure was created with the support of BioRender.com.

In conclusion, this study is the first to show that F-CSA could possess anti-inflammatory properties by suppressing the JNK/NF-κB signaling pathway in LPS-induced RAW 264.7 cells. Additionally, it demonstrated an antiseptic effect by decreasing the IL-6 levels in the blood of mice with LPS-induced septic shock (Figure 8). Therefore, based on the modulating effects of our F-CSA on inflammatory responses in vitro and in vivo, it has development value as a potential therapy.

## Figures and Tables

**Table 1:** The extraction yield of six CL samples.

Group ID	Sample	Used Part	Cultivation	Fermentation	Yield
F-CSA	*C. lanceolata* sprout	Aerial	SMART-Farm	Yes	10.92%
F-CSR	*C. lanceolata* sprout	Roots	SMART-Farm	Yes	15.60%
CSA	*C. lanceolata* sprout	Aerial	SMART-Farm	None	23.86%
CSR	*C. lanceolata* sprout	Roots	SMART-Farm	None	27.30%
CSW	*C. lanceolata* sprout	Whole	SMART-Farm	None	12.96%
CLR	*C. lanceolata*	Roots	Outdoor	None	15.60%

**Table 2:** The calibration curves for luteolin and kaempferol.

Compound	Retention Time	Calibration Equation	Correlation Factor (*r*^2^*)*
Luteolin	24.6	Y = 14,554X − 3681	0.9990
Kaempferol	28.1	Y = 17,480X − 76,444	0.9998

Y = peak area; X = concentration of standards (µg/mL). *r*^2^ = correlation coefficient for five calibration data points (*n* = 3).

**Table 3:** Primer sequences used in RT–PCR analysis.

Target Gene	Primer Sequence	
*iNOS*	F	5′-CAT GCT ACT GGA GGT GGG TG-3′
	R	5′-CAT TGA TCT CCG TGA CAG CC-3′
*COX-2*	F	5′-TGC TGT ACA AGC AGT GGC AA-3′
	R	5′-GCA GCC ATT TCC TTC TCT CC-3′
*IL-6*	F	5′-GAG GAT ACC ACT CCC AAC AGA CC-3′
	R	5′-AAG TGC ATC ATC GTT GTT CAT ACA-3′
*IL-1β*	F	5′-ACC TGC TGG TGT GTG ACG TT-3′
	R	5′-TCG TTG CTT GGT TCT CCT TG-3′
*TNF-α*	F	5′-CTG ATG AGA GGG AGG CCA TT-3′
	R	5′-AGC ACA GAA AGC ATG ATC CG-3′
*β-Actin*	F	5′-ATC ACT ATT GGC AAC GAG CG-3′
	R	5′-TCA GCA ATG CCT GGG TAC AT-3′

**Table 4:** Total phenolic, tannin, flavonoid, and saponin contents of six CL samples.

Phytochemical Tests	F-CSA	F-CSR	CSA	CSR	CSW	CLR
Total phenolic (mg GAE/g)	57.00 ± 1.80	22.35 ± 1.20	6.44 ± 0.48	31.27 ± 0.44	2.31 ± 0.37	10.81 ± 0.51
Total tannin (mg GAE/g)	36.27 ± 1.61	13.66 ± 1.11	4.74 ± 0.95	22.97 ± 0.40	2.35 ± 0.25	7.49 ± 0.22
Total flavonoid (mg CE/g)	26.17 ± 16.40	N.D.	24.57 ± 11.21	N.D.	N.D.	N.D.
Total saponin (mg DE/g)	183.56 ± 0.96	167.59 ± 5.25	156.89 ± 2.95	212.67 ± 3.53	182.04 ± 11.11	216.0 ± 20.38

F-CSA; Fermented *C. lanceolata* sprout aerial part, F-CSR; Fermented *C. lanceolata* sprout root zone, CSA; *C. lanceolata* sprout aerial part, CSR; *C. lanceolata* sprout root zone, CSW; *C. lanceolata* sprout whole plant, CLR; *C. lanceolata* root zone.GAE; gallic acid equivalent, CE; catechin equivalent, DE: diosgenin equivalent. N.D.: not determined.

**Table 5:** Identification of 17 compounds in F-CSA detected by UPLC-ESI-Q/TOF-MS.

	RT (min)	Tentative Identification	Formula	*m*/*z* [M − H]^−^	Mass Error (ppm)	Fragment (*m*/*z*)
1	0.46	Hexitol	C_6_H_14_O_6_	181.0714	−2.1	-
2	0.61	Malic acid	C_4_H_6_O_5_	133.0142	−0.3	89.0249, 115.0038
3	1.90	Kaempferol-3-O-β-D-glucopyranoside	C_21_H_20_O_11_	447.0935	0.5	285.0412
4	2.53	Chrysoeriol-7-O-β-D-glucuroside	C_22_H_22_O_11_	461.1086	−0.6	284.0330, 299.0566
5	3.15	Luteolin	C_15_H_10_O_6_	285.0402	−0.8	133.0295, 151.0038
6	3.70	Kaempferol	C_15_H_10_O_6_	285.0405	0.1	133.0297, 151.0039
7	4.57	Diosmetin	C_16_H_12_O_6_	299.0563	0.5	284.0332
8	5.06	Lancemaside A	C_57_H_90_O_26_	1189.5680	2.8	647.3812
9	6.53	Caryocaroside II-5	C_36_H_56_O_10_	647.3806	0.8	-
10	8.55	Caryocaroside II-5	C_36_H_56_O_10_	647.3801	0.1	-
11	8.74	(*E,E*)-9-Oxooctadeca-10,12-dienoic acid	C_18_H_30_O_3_	293.2117	−1.8	-
12	9.45	Coronaric acid	C_18_H_32_O_3_	295.2279	0.2	-
13	10.91	Linoleic acid	C_18_H_32_O_2_	279.2328	−0.7	-
14	11.84	α-Linolenic acid	C_18_H_30_O_2_	277.2173	−0.1	-
15	11.96	n-Pentadecanal	C_15_H_30_O	271.2279	0.0	-
16	12.72	Linolic acid	C_18_H_32_O_2_	279.2330	0.1	-
17	13.52	Hexadecanoic acid	C_16_H_32_O_2_	255.2328	−0.5	-

**Table 6:** Contents of luteolin and kaempferol in the six CL samples.

Sample	Luteolin (mg/g)	Kaempferol (mg/g)
F-CSA	7.83 ± 0.16	1.09 ± 0.07
F-CSR	N.D.	N.D.
CSA	1.71 ± 0.36	1.65 ± 0.27
CSR	N.D.	N.D.
CSW	tr	tr
CLR	N.D.	N.D.

Luteolin and kaempferol were quantitatively analyzed using HPLC. The concentrations of luteolin and kaempferol were determined using calibration curves generated from standard solutions and the respective equations. F-CSA: Fermented *C. lanceolata* sprout aerial part; F-CSR: Fermented *C. lanceolata* sprout root zone; CSA: *C. lanceolata* sprout aerial part; CSR: *C. lanceolata* sprout root zone; CSW: *C. lanceolata* sprout whole plant; CLR: *C. lanceolata* root zone; N.D.: not detected; tr: trace.

## Data Availability

Not applicable.

## Abbreviations

CL, *codonopsis lanceolata*; COX-2, cyclooxygenase-2; ERK, extracellular signal-regulated kinase; ESI, electrospray ionization; F-CSA, fermented *Codonopsis lanceolata* sprout aerial part extract; F-CSR, fermented *Codonopsis lanceolata* sprout root zone extract; CSA, *Codonopsis lanceolata* sprout aerial part extract; CSR, *Codonopsis lanceolata* sprout root zone extract; CSW, *Codonopsis lanceolata* sprout whole plant extract; CLR, *Codonopsis lanceolata* root zone extract; IL-1β, interleukin-1β; IL-6, interleukin-6; iNOS, inducible nitric oxide synthase; JNK, c-JUN-terminal kinase; LPS, lipopolysaccharide; MAPK, mitogen-activated protein kinase; NF-κB, nuclear factor-kappa B; NO, nitric oxide; NSAIDs, nonsteroidal anti-inflammatory drugs; PGE_2_, prostaglandin E_2_; QTOF, quadrupole time-of-flight; ROS, reactive oxygen species; TLR4, toll-like receptor 4; TNF-α, tumor necrosis factor-α.
